# Comparison of Debris Extrusion and Preparation Time by Traverse, R-Motion Glider C, and Other Glide Path Systems in Severely Curved Canals

**DOI:** 10.1155/ijod/6619901

**Published:** 2025-02-25

**Authors:** Taher Al Omari, Layla Hassouneh, Khawlah Albashaireh, Alaa Dkmak, Rami Albanna, Ali Al-Mohammed, Ahmed Jamleh

**Affiliations:** ^1^Department of Conservative Dentistry, Faculty of Dentistry, Jordan University of Science and Technology, Irbid, Jordan; ^2^Department of Restorative Dentistry, College of Dental Medicine, University of Sharjah, Sharjah, UAE

**Keywords:** debris extrusion, HyFlex EDM Glidepath, One G, R-Motion Glider C, R-Pilot, Traverse

## Abstract

This study evaluated debris extrusion by Traverse and R-Motion Glider C during glide path preparation. Seventy-five mesial roots of mandibular molars with severely curved mesiobuccal canals were distributed into five groups: Traverse, R-Motion Glider C, HyFlex EDM Glidepath, One G, and R-Pilot. During the glide path preparation, debris extruded beyond the apex were collected. The debris weight was calculated (in mg), and the preparation time was recorded (in s). All the systems extruded debris beyond the apex; Traverse extruded the highest amount followed by HyFlex EDM Glidepath, R-Motion Glider C, One G, and R-Pilot (*p*  < 0.01). Traverse required almost half the duration of HyFlex EDM Glidepath to prepare the canals (*p*  < 0.01), while R-Motion Glider C required the least time (*p*  < 0.05). Traverse extruded the highest amount of debris, whilst R-Motion Glider C extruded similar amounts of debris to HyFlex EDM Glidepath, One G, and R-Pilot systems.

## 1. Introduction

The canal anatomy in multirooted teeth with varying curvatures at different levels could be challenging during root canal treatment. Nonetheless, with continuous innovation and development in the manufacturing of nickel titanium (NiTi) rotary files and techniques, the original anatomy of such a canal can be maintained while reducing file fracture and preparation mishaps [1, 2]. Furthermore, with glide path preparation, the risk of creating canal transportation and taper lock of the subsequent shaping files could be further reduced [1, 2].

The glide path preparation is defined as the creation of a smooth and patent passage from the canal orifice to the major foramen. It is prepared with manual or engine-driven small files [1]. Recently, several companies have introduced different NiTi glide path files with greater flexibility and improved cross-sectional design that simplify and enhance the process of establishing the glide path with fewer procedural errors and preparation time [2].

Traverse (KaVo Kerr, CA, USA) is a new rotary glide path NiTi file made with variable heat treatment technology. Two files are available with a taper of 6% and tip diameters of 0.13 and 0.18 mm. The files are made with a triangular cross-section for better cutting efficiency and greater flexibility and strength [3].

R-Motion Glider C (FKG Dentaire, Switzerland) is another recently introduced heat-treated glide path NiTi file that works in a reciprocating motion. The file has a round triangular cross-section with a 0.15-mm tip diameter and 3% taper. The manufacturer claims that this file has improved cyclic fatigue resistance, higher flexibility, and lower screwing effect compared to traditional reciprocating files [4].

Hyflex EDM Glidepath (Coltène/Whaledent, Altstätten SG, Switzerland) is a rotary heat-treated NiTi file that is manufactured from controlled memory (CM) wire and subjected to electric discharges, leading to shaping via melting and vaporization. It has a tip diameter of 0.10 mm and a taper of 5%. The file is designed with different cross-sectional shapes, which are quadratic in the apical part and trapezoidal in the coronal part of the instrument [5]. The unique manufacturing method and design of the file have been shown to enhance cyclic fatigue resistance, phase transformation temperatures, and hardness [5, 6].

One G file (MicroMega, Besancon, France) is a conventional rotary NiTi glide path file. It has a tip diameter of 0.14 mm and a file taper of 3%. It is designed with asymmetric cross-section, three cutting blades, and varying pitch lengths which collectively reduce the file engagement inside the canals and allow efficient debris removal [7].

R-Pilot (VDW, Munich, Germany) is a reciprocating NiTi glide path file made of M-Wire alloy. The file has an S-shaped cross-section with a tip diameter of 0.125 mm and a file taper of 4% [8].

During the chemomechanical preparation step in root canal treatment, undesirable extrusion of pulpal tissue remnants, dentinal chips, microorganisms, and irrigation solutions into the periradicular tissue may occur [9]. These extrusions may lead to delayed periapical recovery, inflammation, flare-ups, and postoperative pain which in turn may compromise the prognosis of endodontic treatment [9, 10].

The amount of extruded debris can be affected by several factors including the anatomy of teeth, access cavity preparation, irrigation technique and temprature, type of file, file's motion kinematics, type of treatment, and preparation techniques [10–16]. It has been agreed in literature that creating glide path using rotary files produced lesser amounts of debris extrusion compared to manual stainless-steel files [17, 18]. Therefore, in this study, glide path engine-driven files with different geometries and cross sections used in different speeds and torques and motion kinematics were used. To the best of our knowledge, there are no published data available regarding the effect of Traverse and R-Motion Glider C as glide path files on the apical extrusion of debris in severely curved canals. Hence, the current in vitro study aimed to compare debris extrusion and preparation time for Traverse, R-Motion Glider C, and other glide path systems in severely curved canals. The null hypothesis was that the amount of extruded debris and preparation time showed no significant difference between the tested glide path files.

## 2. Methodology

### 2.1. Sample Preparation

This research was approved by the Institutional Research Ethics Committee at Jordan University of Science and Technology (IRB/23/2017). Mandibular first molar teeth extracted for periodontal reasons were selected from a pool of teeth in the university hospital after obtaining patients' consent to be used for research purposes.

The teeth were examined clinically and radiographically (periapical radiographs were taken in the buccolingual and mesiodistal aspects) to include intact teeth with severely curved mesiobuccal canals in the range of 20°–40° as measured using image analysis software (AxioVision 4.5; Carl Zeiss Vision, Hallbergmoos, Germany). Any tooth with previously treated root canals, external defects, straight or extremely curved canals, calcified or wide canals, open apices, or other anatomic irregularities was excluded.

Based on a pilot study, the sample size was calculated at an 80% power and 95% confidence level to detect a minimum extruded debris weight difference of 0.3 mg between the experimental groups. The common standard deviation was set as 1.5 mg within a group. The results indicated that each group should be composed of 15 teeth. Thus, a total of 75 mandibular molars were chosen, numbered, and randomly divided into five groups according to the type of file used (www.random.org).

The calculus and soft tissue remnants on the root surface were cleaned carefully with ultrasonic tips, and the teeth were stored inside a glass jar full of physiological saline solution until use.

Access cavity with high-speed diamond bur under water cooling was performed. The mesial root was separated, and the mesiobuccal root canal was allocated, irrigated with 2.5% sodium hypochlorite, and enlarged to size 10 file. The canal was excluded if its width near the apex was larger than a size 10 file. Then, occlusal reduction was made with low-speed diamond saw to standardize the root length to 19 mm.

### 2.2. Debris Collection

**Figure 1:**
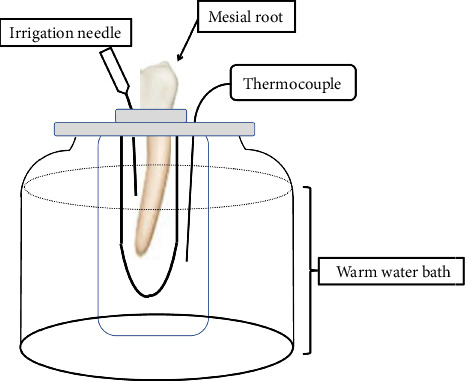
Schematic drawing of the experimental setup.

The debris collection device was adopted from a previous work of Myers and Montgomery [19] with some modifications. Briefly, glass tubes with 10 mL volume were weighed five times by using an electronic balance (Citizen CX 220 Analytical Lab Balance, Internal Cal. Weighing Hook, USA) (accuracy of 10^−5^ g), and their mean values were recorded. Then, each mesial root was fixed inside the tube in a standing position where the coronal part was exposed above the tube opening level to ensure no fluid leakage. The root was covered with silicon index until the decoronated level with cavity maintained for irrigation and tested for fluid leakage by flooding the access cavity with distilled water and monitoring the fluid for 1 min. In case of fluid leakage, another silicon index was made with better tooth adaptation and a tight seal. Afterward, a 27-G irrigation needle was inserted in the silicon index to equalize pressure. Then, the glass tube was covered with aluminum foil and inserted into a larger dry glass vial that was submerged in a warm water bath (37°C) as confirmed with a thermocouple (MN35, Digital Mini MultiMeter, Boston, Massachusetts, USA) (Figure 1).

### 2.3. Treatment Protocol

The working length (WL) was set 1 mm short of the root length, and the samples were prepared using an endodontic motor (VDW Silver, VDW, Munich, Germany) according to the manufacturers' recommendations as follows: Continuous motion was adopted for groups Traverse, HyFlex EDM Glidepath, and One G using files sizes 13/06, 10/05, and 14/03, respectively. The rotational speeds/torques were set as 500/1.5 Ncm, 300/1.8 Ncm, and 400/1.2 Ncm, respectively. “Reciproc ALL” mode was adopted for R-Motion Glider C (15/03) and R-Pilot (12.5/04) glide path files.

Each file was used to prepare one canal with three pecking strokes in 3-mm amplitude. The file was withdrawn, cleaned, inspected for defects, and then used until reaching the WL. The canal was irrigated with a total of 5 mL of 2.5% sodium hypochlorite using a side-vented needle under high vacuum suction throughout the procedure. The needle was bent and marked 2 mm short of the WL by using a rubber stopper. A final rinse with 1 mm distilled water was made externally at the apex surface inside the tube before replacing the sample. Then, the sample was taken out and maintained inside an incubator for 2 weeks at 70°C to complete dryness. Afterward, the glass tubes were critically weighted five times, and the mean value was calculated. Then, the weight of the extruded debris was calculated by subtracting the weight measured before the preparation from that measured after the preparation. The preparation time was recorded using a digital chronometer which included the active preparation, file change, file cleaning, and canal irrigation. The preparation was performed by an endodontist using magnifying loupes at 3.5× magnification.

### 2.4. Statistical Analysis

Shapiro–Wilk test indicated that the data are not normally distributed (*p*  < 0.001). Therefore, differences between the tested systems in terms of weight of extruded debris (mg) and preparation time (s) were analyzed using Kruskal–Wallis test and Mann–Whitney *U* test. Linear regression was used to analyze statistically the incidence of debris extrusion by tested system and preparation time. All analyses were performed using Statistical Package for Social Sciences software (Version 23, IBM SPSS Statistics, USA) at 5% significance level.

## 3. Results

Table 1 shows the results of the apical extrusion of debris and preparation time for each tested system. Linear regression analysis revealed no significant effect of the tested system and preparation time on extruded debris. All the systems extruded debris beyond the apex; Traverse extruded the highest amount of debris which was significant than other groups (*p*  < 0.05). HyFlex EDM Glidepath, R-Motion Glider C, One G, and R-Pilot had comparable extrusions (*p* > 0.05). The HyFlex EDM required almost double the duration of other systems to prepare the canals (*p*  < 0.01), while R-Motion Glider C required the least time which was significantly lower than Traverse (*p*=0.026) but comparable to One G and R-Pilot (*p* > 0.05).

## 4. Discussion

Clinically, eliminating bacteria and their by-products while maintaining the original configuration of root canals is the main objective of canal cleaning and shaping. During canal preparation, the file acts as a plunger introduced apically which might push irrigants and intracanal contents beyond the apex. This might cause postoperative pain and involve the host immune response against extruded debris containing necrotic pulp tissues and bacteria or obturating material which may trigger inflammatory reactions in the periapical region [9]. A glide path concept is introduced to facilitate canal preparation with shaping files [20]. Moreover, it has shown to reduce fatigue on shaping instruments and ultimately increase their life span [21]. A previous study showed that glide path improved the centering ability in the apical part of S-shaped canals, regardless of the tested motion kinematics [22]. However, past studies reported that all tested instrumentation techniques and file systems including the glide path preparation seem to force intracanal debris into the periapical tissues to varying extents [23, 24]. Nevertheless, a randomized clinical trial stated that glide path preparation prior to instrumentation resulted in significantly less postoperative pain [25]. Therefore, the performance of glide path files in curved canals has been investigated.

The extrusion of debris by newly released glide path files should be studied to enhance patient satisfaction and improve treatment success. This study evaluated the apical extrusion of different glide path single-file instruments as well as the time necessary to prepare the canals. It presented the effects of many factors such as file size and taper, cutting efficiency, rotational speed, cross-sectional design, and motion kinematics. Apically extruded debris was evident in all the tested groups. The smallest amount of apical debris extrusion was observed in R-Pilot glide path file, while the largest amount was observed in Traverse file. R-Motion Glider C required the least preparation time. Thus, the null hypothesis as rejected.

A greater taper of the file might cause more debris extrusion [26]. For instance, the increased weight of debris extruded with Traverse file might be due to its large taper. One study showed that a larger taper resulted in higher debris extrusion because of the greater preparation into the dentinal walls [27]. In this study, Traverse and HyFlex EDM Glidepath extruded more debris than R-Motion Glider C, One G, and R-Pilot.

In terms of file design, the triangular cross-sectional shape in Traverse file aids in its cutting efficiency and therefore produces more dentin debris. The R-Pilot file has low core area with two cutting edges and an S-shaped cross-sectional design which enhance its cutting efficiency, pose less stress on dentin, and reduce the time required for canal preparation significantly. This allows the instrument to move without pushing debris apically [28]. Consistently, Kurt, Zengin, and Üstün [29] found R-Pilot had reduced amount of apical debris compared to files with convex, triangular, and parallelogram designs.

The tested files are used with different motion kinematics. In the current study, the reciprocating R-Pilot and R-Motion Glider C files extruded relatively less debris than the other rotary files which is in agreement with a previous study [20]. Other studies found no difference in debris extrusion between rotary and reciprocating files [30, 31], while others indicated that reciprocating motion extruded more debris [32, 33]. These discrepancies could be explained by the design of the files tested. Among the tested glide path files that showed lesser debris extrusion is One G. The reason behind this might be attributed to the lesser taper, asymmetric cross-section, and the off-center design which allow debris removal toward coronal direction [7, 17, 18]. Also, the results seem to suggest that reciprocating glide path files (R-Motion Glider C and R-Pilot) had lower levels of extrusion than rotary glide path files (Traverse, HyFlex EDM Glidepath, and One G). It was reported that glide path files with reciprocating motion had greater cutting efficiency than those with continuous rotation [28].

The weights of extruded debris were generally found to be higher than what are published in the literature [24, 29]. This is consistent to a previous study which found that NaOCl irrigant resulted in significantly more extruded debris than distilled water [12]. The extruded sodium hypochlorite irrigant tends to deposit crystals upon evaporation which will in turn increase the weight of extruded debris [11, 24]. Nonetheless, this was done to mimic the clinical conditions. Besides that, all the samples were irrigated with the same volume (a total of 5 mL) during the preparation of each canal.

Severely curved canals for which size 10 K-file reached the WL with resistance were selected. The WL was determined to be 1 mm short of the canal length since preparing root canal at or beyond the apical foramen extrudes more debris [34]. The occlusal surface of each tooth was reduced to adjust the WL to 18 mm to standardize the preparation and irrigation penetration depths. With these, the selected canals were identified as severely curved and narrow canals with standardized lengths. Therefore, these parameters were confirmed to purely study the effect of glide path preparation with different file systems on debris extrusion.

This in vitro study has certain limitations. The periapical pressure that might act as a natural barrier to minimize apical extrusion is lacking. Placing the floral sponge at the apex has been suggested to simulate the periapical barrier [35]. However, this approach revealed dispersion in the calculated debris since the sponge might absorb the debris and irrigant [35]. Thus, the current study was done without barrier according to Myers and Montgomery [19], and the current results should be translated to clinical practice with caution. Side-vented needles were used in all the groups to reduce the influence of needle design on irrigant extrusion beyond the apex.

The extrapolation of current findings to clinical conditions should be made with caution. Drying debris at 70°C may not reflect clinical settings, and using canals with severe curvature could restrict applicability to cases with moderate or mild curvature. Nonetheless, this model was utilized by previous studies [14, 15, 19, 36] which could provide controlled conditions for reliable comparison between the tested groups. Although glide path preparation with the tested systems caused measurable debris extrusion beyond the apex, R-Pilot extruded the least amount of debris in severely curved canals. Further in vivo investigations are needed to investigate effect of apically extruded debris on endodontic treatment success.

## 5. Conclusions

Glide path preparation caused measurable debris extrusion beyond the apex in severely curved canals, regardless of the file system used. R-Motion Glider C, One G, and R-Pilot exhibited favorable outcomes in terms of debris extrusion and preparation time.

## Figures and Tables

**Table 1:** Mean (SD) and median of the weight of apically extruded debris (mg) and preparation time (s) in the tested systems (*n* = 15).

Tested system	Apically extruded debris		Time	
	Mean (SD)	Median	Mean (SD)	Median
Traverse	6.59 (2.73)	7.33^a^	52.87 (15.45)	50^a^
R-Motion Glider C	3.71 (1.67)	3.16^b^	41.33 (10.54)	39^b^
HyFlex EDM Glidepath	4.52 (2.49)	3.96^b^	102.13 (48.52)	83^c^
One G	3.43 (1.40)	3.19^b^	45.00 (6.43)	42^a,b^
R-Pilot	3.06 (1.44)	2.48^b^	49.73 (15.17)	48^a,b^

*Note:* Different superscript letters indicate statistical significance.

## Data Availability

The data underlying this article will be shared upon reasonable request from the corresponding author.
